# Right Ventricular Dysfunction in Lupus Patients With Pulmonary Hypertension

**DOI:** 10.5935/abc.20180125

**Published:** 2018-07

**Authors:** Silvio Henrique Barberato

**Affiliations:** Cardioeco – Centro de Diagnóstico Cardiovascular, Curitiba, PR – Brazil

**Keywords:** Ventricular Dysfunction, Right, Lupus Erythematosus, Cardiovascular Diseases, Lung Diseases, Hypertension, Pulmonary, Echocardiography, Strain, Strain Rate

The importance of the right ventricle in cardiovascular physiology has been underestimated for decades. Previously considered a mere conduit, the right ventricle is currently known to play a major role in maintaining global cardiac function intact. In parallel, right ventricular (RV) systolic function has been shown to be an essential determinant of clinical outcomes in several scenarios,^1^ and should thus be considered in the individualized management of patients. The need to diagnose RV dysfunction is evident. Because of its wide availability, echocardiography is the most frequently used imaging test in clinical practice to assess RV size and function. That assessment can be hindered by the complex RV anatomy; thus, important international societies of cardiovascular imaging have recommended the routine and systematic addition of several echocardiographic measurements and techniques.^2,3^ That approach includes conventional parameters, such as RV basal diameter (normal ≤ 41 mm) and tricuspid annular plane systolic excursion (TAPSE - normal ≥ 17 mm), as well as advanced parameters, such as the *s* wave of the RV free wall on tissue Doppler (normal ≥ 9.5 cm/s), ejection fraction on 3D echocardiography (normal ≥ 45%) and longitudinal strain of the RV free wall (normal ≥ -20%).

In this scenario, strain (systolic shortening percentage) and strain rate (shortening rate), calculated by speckle tracking on two-dimensional echocardiography (2D speckle tracking or 2D-STE), emerge as alternatives in the RV systolic function analysis. The longitudinal strain of the RV free wall, excluding the ventricular septum, showed prognostic value in patients with signs and symptoms of cardiopulmonary disease, such as heart failure, myocardial infarction, pulmonary hypertension, congenital heart diseases, RV arrhythmogenic cardiomyopathy and amyloidosis.^1^ Right ventricular longitudinal strain is a parameter less dependent on the angle, with less intra- and interobserver variability, that can apparently detect early RV dysfunction. Its drawbacks include the high dependence on image quality and the variability of the software of the equipment available in the market.^3^ Recently an international consensus has been reached to standardize the use of 2D-STE to obtain RV strain.^4^ The specific use of right-ventricle-focused apical 4-chamber view is recommended for correct strain measurement. Extreme care should be taken to define the region of interest (ROI) of the endocardial border (suggested ROI: 5 mm), because of the RV shape and thin walls. The pericardium should be excluded from the analysis, because of the risk of strain underestimation.

The study by Luo et al.,^5^ published in this issue of the *Arquivos Brasileiros de Cardiologia*, shows that the assessment of strain and strain rate by use of 2D-STE can detect early RV dysfunction in individuals with systemic lupus erythematosus (SLE) associated with mild and subclinical pulmonary hypertension [PH: systolic pulmonary artery pressure (SPAP) between 30 and 50 mmHg]. It is worth noting that, considering the other conventional and nonconventional parameters of RV size and systolic function, RV dysfunction was only diagnosed in individuals with moderate to severe PH (SPAP ≥ 50 mmHg). It is necessary to acknowledge the little methodological limitation in estimating right atrial pressure, to which only two values were attributed (8 or 15 mmHg) in the dynamic analysis of the inferior vena cava. This might have overestimated the SPAP in some patients, but that bias does not invalidate the results of that study. Another study has reported that the survival rates of patients with SLE who develop PH seem lower than those of individuals with primary PH.^6^ The findings of the study by Luo et al.^5^ allow us to speculate that RV dysfunction is the mediator of the high mortality risk in that group of individuals. Those findings suggest that the use of strain to analyze RV systolic function in SLE can select patients in a subclinical phase who require careful surveillance and early therapy to prevent the development of RV failure and cardiovascular complications. Further studies are necessary to deepen the pathophysiological knowledge of RV dysfunction in the clinical context of SLE and to assess the role of intervention strategies to reduce mortality.
